# Pediatric obesity in the United States: Age–period–cohort analysis

**DOI:** 10.1016/j.heliyon.2024.e32603

**Published:** 2024-06-08

**Authors:** Ashley W. Kranjac, Dinko Kranjac, Roxanne I. Aguilera

**Affiliations:** a Department of Sociology, Wilkinson College, Chapman University, Orange, CA, USA; b The Earl Babbie Research Center, Chapman University, Orange, CA, USA; c Psychology Program, College of Health and Community Well-Being, University of La Verne, La Verne, CA, USA; d Institute of Mental Health and Psychological Well-Being, University of La Verne, La Verne, CA, USA

## Abstract

The rates of obesity among American children aged 2–5 years has reached a historic high. It is crucial to identify the putative sources of population-level increases in obesity prevalence among preschool-aged children because early childhood is a critical window for obesity prevention and thus reduction of future incidence. We used the National Health and Nutrition Examination Survey data and hierarchical age–period–cohort analysis to examine lifecycle (i.e., age), historical (i.e., period), and generational (i.e., cohort) distribution of age- and sex-specific body mass index z-scores (zBMI) among 2–5-year-olds in the U.S. from 1999 to 2018. Our current findings indicate that period effects, rather than differences in groups born at a specific time (i.e., cohort effects), account for almost all of the observed changes in zBMI. We need a broad socioeconomic, cultural, and environmental strategy to counteract the current obesogenic environment that influences children of all ages and generations in order to reach large segments of preschoolers and achieve population-wide improvement.

## 1 Introduction

According to data from the National Health and Nutrition Examination Survey (NHANES), the rate of obesity among children aged 2–5 years old in the United States has nearly tripled over the last five decades [1]. Specifically, the overall prevalence of obesity for this age group increased from 5 % in 1971/1974 to 13.9 % in 2003/2004, followed by a decrease to 8.4 % in 2011/2012 and a rebound to 13.4 % in 2017/2018 [1]. This enduring epidemic is a major public health challenge not only because of pediatric obesity-associated comorbidities but also because of sustained obesity and obesity-related complications into adolescence and adulthood [[2], [3], [4]]. Unintentional, excessive weight gain at the *individual* level develops from a chronic positive energy balance through an interplay of genetic, biological, behavioral, socioeconomic, and environmental factors but the root causes of the changing *population*-level trends in pediatric obesity prevalence remain largely unknown [[5], [6], [7], [8], [9], [10]]. It is crucial to identify the putative sources of population-level increases in pediatric obesity prevalence because early childhood is a critical window for obesity prevention and thus reduction of future incidence [11]. The observed population-level patterns in pediatric obesity might be a consequence of unique, time-varying contributors such as *age* (association between age and weight status) and/or *period* (the date when weight status is assessed regardless of age and birth year) and/or *cohort* (changes in weight status among groups of individuals born in different cohorts) [1,[12], [13], [14]]. Specifically, age effects embody variations caused by age-related physiological and developmental changes [12,13]. Period effects reflect broader social, cultural, economic, and environmental changes that are unique to time periods and create similar obesogenic contexts for children of all ages (e.g., public health policies or medical technology) [12,13]. Cohort effects represent individual exposure and formative experience during the child's lifetime [12,13].

Vis-à-vis individual-level indicators, the prevalence of pediatric obesity is associated with a child's age, sex, and race/ethnicity [[15], [16], [17], [18]]. Indeed, age-related body mass index (BMI) data indicate that the rates of obesity increase with increasing age [16]. Notably, 50 % of 2-year-olds with obesity return to a healthy weight in adolescence whereas 75%–90 % of 3–4-year-olds with obesity will have obesity in adolescence [4,17]. Further, within populations, Black, Indigenous, and People of Color (BIPOC) children are disproportionally burdened by obesity, but this association is complicated by the influence of household income and education [[19], [20], [21], [22]]. Important for our current study, these individual-level factors are associated with health behaviors including overall levels of physical activity/sedentary behaviors (e.g., screen time) and dietary patterns (e.g., consumption of high-caloric, energy-dense foods with little-to-no nutritional value) [23,24]. Certainly, consumption of sugar-sweetened beverages and higher intake of total protein is associated with higher childhood BMI or BMI z-scores (zBMI) [25,26]. Additionally, mother's age at childbirth, cigarette smoking during pregnancy, and early-life breastfeeding practices influence the risk of child obesity [[27], [28], [29], [30], [31]]. These maternal factors influence infant birth weight, which is independently associated with subsequent BMI status [31]. Crucially, current outcomes for individual-level interventions (e.g., lifestyle and behavioral modifications) to prevent pediatric obesity are relatively modest [32]. With that in mind, it is urgent to identify the source of *population*-level increases in childhood BMI if we are to reach a consensus about how to “move the needle” on attaining the *Healthy People 2030* pediatric obesity target [33,34]. In fact, the U.S. has never met the *Healthy People 2020* goal of 9.6 % obesity prevalence among children aged 2–5 years old [16,35]. Thus, to disentangle the population-level drivers of obesity, in this paper we used the NHANES data and hierarchical age–period–cohort (HAPC) analysis to examine lifecycle (i.e., age), historical (i.e., period), and generational (i.e., cohort) distribution of age-specific and sex-specific zBMI among 2–5-year-olds in the U.S. over the last two decades [13,14]. To the best of the authors' knowledge, we are the first to investigate obesity trends among American preschoolers using HAPC analysis.

## 2 Methods

### 2.1 Data

We analyzed the NHANES cross-sectional data from ten 2-year “continuous” cycles (1999–2018) [36]. The sample is representative of the U.S. civilian, noninstitutionalized population [36]. We restricted the sample to 2–5-year-olds because life-course trajectory of body weight gain is established during the preschool years [11,37]. Listwise deletion of missing values yielded a final sample size of 6234.^1^ There is no statistically significant difference between the full sample and our final sample after exclusion due to missing values (*t* = 1.1872, *p* = 0.2352). We applied the NHANES cycle-specific sampling weights that account for differences in the unequal probabilities of selection and non-response [36].

### 2.2 Measures

The dependent variable is age-specific and sex-specific BMI (weight (kg)/height (m) [2]) that we transformed into z scores (zBMI) based upon the widely used *2000 Centers for Disease Control and Prevention (CDC)* BMI-for-age *Growth Charts for the United States* [38,39]. The body measure data (i.e., height and weight) were collected objectively by trained health technicians [36]. We included covariates to represent child intrauterine environment, as well as demographic, nutrition, physical activity, family, and socioeconomic factors known to associate with body weight status. The operational definitions and descriptive statistics of all variables included in the analysis are available in Table 1.

**Table 1:** Summary Weighted Statistics for All Variables in the Analysis among Children Aged 2–5 years old, 1999–2018 NHANES (N = 6234).

Dependent Variable		Mean or %	SD	Min	Max	
	BMI Z-scores	−0.03	1.00	−2.52	8.57	
Level-1 Variables						
Age	Respondent's age at survey year	3.52	1.14	2	5	
	Centered around grand mean					
Sex	Respondent's sex: 1 = girl; 0 = boy	51 %	0.50	0	1	
Race/Ethnicity	Respondent's race/ethnicity					
Non-Hispanic White	1 = white	58 %	0.46	0	1	
Non-Hispanic Black	2 = black	13 %	0.44	0	1	
Hispanic	3 = hispanic	22 %	0.47	0	1	
Other Race	4 = other	7 %	0.28	0	1	
Maternal Age	Respondent's mother's age at birth:					
Maternal Age 1	1 = 14–19 years	10 %	0.34	0	1	
Maternal Age 2	2 = 20–35 years	79 %	0.42	0	1	
Maternal Age 3	3 = 36+ years	11 %	0.29	0	1	
Pregnant Smoking	Respondent's mothers' smoking status:					
	1 = not a pregnant smoker; 0 = otherwise	14 %	0.34	0	1	
Birthweight	Respondent's weight at birth					
Birthweight 1	1 = < 5 pounds	6 %	0.21	0	1	
Birthweight 2	2 = ≥ 6 and ≤8 pounds	84 %	0.40	0	1	
Birthweight 3	3 = ≥ 9 pounds	10 %	0.25	0	1	
Breastfed	Respondent's breastfeeding status:					
	1 = not breastfed; 0 = otherwise	72 %	0.47	0	1	
Energy	Respondent's age/sex-specific daily caloric intake (kcals)					
	1 = above recommended calories; 0 = otherwise	50 %	0.50	0	1	
Fat	Respondent's age/sex-specific daily fat intake (gm)					
	1 = above recommended fat; 0 = otherwise	69 %	0.46	0	1	
Protein	Respondent's age/sex-specific daily protein intake (gm)					
	1 = above recommended protein; 0 = otherwise	50 %	0.50	0	1	
Physical Activity	Respondent's number of days physically active last week					
Activity 1	1 = ≤ 2 days	48 %	0.50	0	1	
Activity 2	2 = ≥ 3 days	13 %	0.33	0	1	
Activity 3	3 = ≥ 6 days	39 %	0.48	0	1	
Income	Respondent's parents' household income in 2018 dollars (thousands)	$74,210	$56,650	$2500	$186,580	
Food Secure	Respondent's households' food security status:					
	1 = Food Insecure; 0 = otherwise	20 %	0.44	0	1	
Level-2 Variables			N	Min	Max	
Period	Survey Year		10	1999	2018	
Cohort	Five-Year birth cohort		4	1995	2016	

Note: Age: Median, 3; Interquartile Range (IQR), 2–4. Income: Median $32,399; IQR, $17,499-$67,499.

### 2.3 Statistical analysis

We estimate age, period, and cohort effects on children's zBMI using a novel HAPC modeling technique [13,40]. With this method, a two-level mixed (fixed and random) effects model is specified. This approach accounts for the possibility that children in the same survey year and/or cohort group may have similar zBMI simply because they share similar random period and/or cohort error components (i.e., social experiences). Specifically, individuals are nested within cells cross-classified in two social contexts: birth cohorts and survey years. Level-1 (within period-cohort cells) is a fixed effects quadratic estimation for age and other individual-level covariates within each period-by-cohort group. This tells us how much of the change in zBMI is attributable to variation in physiological changes that occur during the lifetime and/or child's intrauterine environment, demographic background, diet, physical activity, family context, and socioeconomic status (SES) net of period and cohort effects. Level-2 (between period-cohort cells) are normally-distributed random effects for period and cohort. This tells us how much of the population-level variation in child zBMI is attributable to changing socioeconomic and/or physical environment that affects outcomes for all children simultaneously (i.e., period effect) *or* changing population composition due to common initial event experience [(e.g., birth year); i.e., cohort effect]. HAPC models are flexible in outcome distributions but, like other APC models, are biased when age, period, and cohort are linear. To break the exact linear dependency between the three explanatory variables (i.e., cohort = period – age) and resolve under-identification, a fundamental methodological challenge in APC analysis, we grouped birth cohorts into commonly used 5-year intervals and, upon verification of a curvilinear relationship, treated zBMI as a quadratic function of age [41].

## 3 Results

In Table 1, we present the operational definitions and descriptive statistics for all variables included in the analysis. Average zBMI in the sample was −0.03 (range −2.52 to 8.57), which roughly translates to within-the-“normal” weight range (mean BMI = 16.43). In Table 2, we display estimates of fixed and random effects coefficients of zBMI from the multilevel models. Model 1 shows a significant quadratic age effect controlling for random period and cohort effects. Adjusting for time period and birth cohort variation, zBMI decreases by 0.42 standard deviations (SDs) with every 1-year increase in age (−0.42; p < 0.001), but the decline increases at the rate of 0.06 SDs with every passing year across the life course (0.06; p < 0.001). In the lower portion of Table 2, we display residual variance components at Level-2. Data indicate that zBMI vary significantly by time period and birth cohort net of the age effect.

**Table 2:** Estimates from cross-classified random effects age-period-cohort models of BMI Z-scores.

Fixed Effects	Model 1				Model 2				Model 3			
	*Coefficient*	*se*	*95 % CI*	*t Ratio*	*Coefficient*	*se*	*95 % CI*	*t Ratio*	*Coefficient*	*se*	*95 % CI*	*t Ratio*
Intercept, π_0_	0.82***	0.18	[0.47, 1.17]	4.54	0.86***	0.18	[0.51, 1.21]	4.67	0.89***	0.18	[0.54, 1.24]	4.87
Age, π_1_	−0.42***	0.11	[-0.64, −0.20]	−3.99	−0.38***	0.10	[-0.58, −0.18]	−3.66	−0.39***	0.10	[-0.59, −0.19]	−3.78
Age [2], π_1_	0.06***	0.02	[0.02, 0.10]	3.61	0.05***	0.02	[0.01, 0.09]	3.21	0.06*	0.03	[0.00, 0.12]	2.01
Girl, π_2_					−0.06*	0.03	[-0.12, −0.00]	−2.09	−0.06*	0.03	[-0.12, −0.00]	−2.08
Race/Ethnicity (ref. = NH White)												
NH Black, π_3_					−0.03	0.04	[-0.11, 0.05]	−0.74	−0.03	0.04	[-0.11, 0.05]	−0.74
Hispanic, π_4_					0.16***	0.04	[0.08, 0.23]	4.52	0.16***	0.04	[0.08, 0.24]	4.47
Other Race, π_5_					−0.18***	0.06	[-0.30, −0.06]	−3.19	−0.18***	0.06	[0.06, 0.30]	−3.21
Maternal Age (ref. = Maternal Age 2: 20–35 years)												
Maternal Age 1 (14–19 years), π_6_					0.06	0.05	[-0.04, 0.16]	1.22	0.06	0.05	[-0.04, 0.16]	1.20
Maternal Age 3 (36+ years), π_7_					0.02	0.05	[-0.08, 0.12]	0.46	0.02	0.05	[-0.08, 0.12]	0.48
Not a Pregnant Smoker, π_8_					−0.15**	0.05	[-0.25, −0.05]	−3.17	−0.15*	0.05	[-0.25, −0.05]	−3.16
Birthweight (ref. = Birthweight 2: ≥6 and ≤8 pounds)												
Birthweight 1 (<5 pounds), π_9_					−0.19**	0.07	[-0.33, −0.05]	−2.75	−0.19**	0.07	[-0.33, −0.05]	−2.79
Birthweight 2 (≥9 pounds), π_10_					0.27***	0.06	[0.15, 0.39]	4.61	0.27***	0.06	[0.15, 0.39]	4.53
Not Breastfed, π_11_					0.12***	0.03	[0.06, 0.18]	3.60	0.10**	0.04	[0.02, 0.18]	2.83
Above Recommended Calories, π_12_					0.03	0.04	[-0.05, 0.11]	0.62	0.03	0.04	[-0.05, 0.11]	0.60
Above Recommended Fat, π_13_					0.04	0.04	[-0.04. 0.12]	1.04	0.05	0.04	[-0.03, 0.13]	1.08
Above Recommended Protein, π_14_					0.09*	0.04	[0.01, 0.17]	2.22	0.09*	0.04	[0.01, 0.17]	2.29
Physical Activity (Physical Activity 3: ref. = ≥ 6 days)												
Physical Activity 1 (≤2 days), π_15_					−0.01	0.06	[-0.13, 0.11]	−0.18	−0.01	0.06	[-0.13, 0.11]	−0.21
Physical Activity 2 (≥3 days), π_16_					−0.01	0.05	[-0.11, 0.09]	−0.27	−0.01	0.05	[-0.11, 0.09]	−0.92
Income					−0.01***	0.00	[-0.01, −0.01]	−3.29	−0.01	0.05	[-0.11, 0.09]	−0.25
Food Insecure, π_21_					−0.01	0.04	[-0.09, 0.07]	−0.15	−0.01	0.04	[-0.09, 0.07]	−0.13
Age × Not Breastfed									0.06*	0.03	[0.00, 0.12]	2.01
***Random Effects***												
**Cohort**	***Coefficient***	***se***	***95 % CI***	***t Ratio***	***Coefficient***	***se***	***95 % CI***	***t Ratio***	***Coefficient***	***se***	***95%CI***	***t Ratio***
1995	0.12	0.23	[-0.33, 0.23]	0.51	0.13	0.22	[-0.30, 0.56]	0.56	0.13	0.22	[-0.30, 0.56]	0.03
2000	0.06	0.19	[-0.31, 0.43]	0.31	0.06	0.19	[-0.31, 0.43]	0.31	0.08	0.19	[-0.29, 0.45]	0.28
2005	0.16	0.15	[-0.13, 0.45]	1.07	0.14	0.15	[-0.15, 0.43]	0.94	0.16	0.15	[-0.13, 0.45]	0.25
2010	0.10	0.11	[-0.12, 0.32]	0.94	0.09	0.11	[-0.13, 0.31]	0.78	0.10	0.11	[-0.12, 0.32]	0.90
**Period**												
1999	−0.32	0.21	[-0.73, 0.09]	−1.51	−0.41^#^	0.21	[-0.82, 0.00]	−1.96	−0.41	0.21	[-0.82, 0.00]	−1.97
2001	−0.26	0.2	[-0.65, 0.13]	−1.30	−0.34	0.19	[-0.71, 0.03]	−1.74	−0.34	0.19	[-0.71, 0.03]	−1.76
2003	−0.13	0.18	[-0.48, 0.22]	−0.72	−0.23	0.18	[-0.58, 0.12]	−1.31	−0.24	0.18	[-0.59, 0.11]	−1.35
2005	−0.22	0.17	[-0.55, 0.11]	−0.27	−0.28	0.17	[-0.61, 0.05]	−1.64	−0.29	0.17	[-0.62, 0.04]	−1.68
2007	−0.29^#^	0.14	[-0.56, −0.02]	−2.01	−0.32*	0.14	[-0.59, −0.05]	−2.26	−0.33	0.14	[-0.60, −0.06]	−2.32
2009	−0.20	0.13	[-0.46, 0.06]	−1.54	−0.22	0.14	[-0.49, 0.05]	−1.63	−0.23	0.14	[-0.50, 0.04]	−1.68
2011	−0.31*	0.11	[-0.53, −0.09]	−2.73	−0.33*	0.12	[-0.57, −0.10]	−2.65	−0.33*	0.12	[-0.56, −0.09]	−2.71
2013	−0.19	0.09	[-0.37, −0.01]	−2.01	−0.19	0.11	[-0.41, 0.03]	−1.73	−0.19	0.11	[-0.41, 0.03]	−1.77
2015	−0.15^#^	0.09	[-0.33, 0.03]	−1.66	−0.14	0.10	[-0.34, 0.06]	−1.37	−0.15	0.10	[-0.35, 0.05]	−1.43
2017	0.32	0.21	[-0.09, 0.73]	1.51	0.41^#^	0.21	[-0.00, 0.82]	1.96	0.41	0.21	[-0.00, 0.82]	1.97
*Variance Components*	***Variance***	***sd***		***p value***	***Variance***	***sd***		***p value***	***Variance***	***sd***		***p value***
Cohort	0.00001*	0.00185		0.003	0.00003*	0.01242		0.032	0.00002*	0.01169		0.033
Period	0.00003*	0.00526		0.037	0.00001*	0.00181		0.033	0.00001*	0.00180		0.033
Individual	0.94603*	0.97264		0.003	0.91206*	0.95502		0.032	0.91130*	0.94387		0.033
**Model Fit**	18831.38				18583.92				18578.22			

Note.

# p < 0.10.

∗ p ≤ 0.05.

∗∗ p ≤ 0.01.

∗∗∗ p ≤ 0.001.

**Fig. 1:**
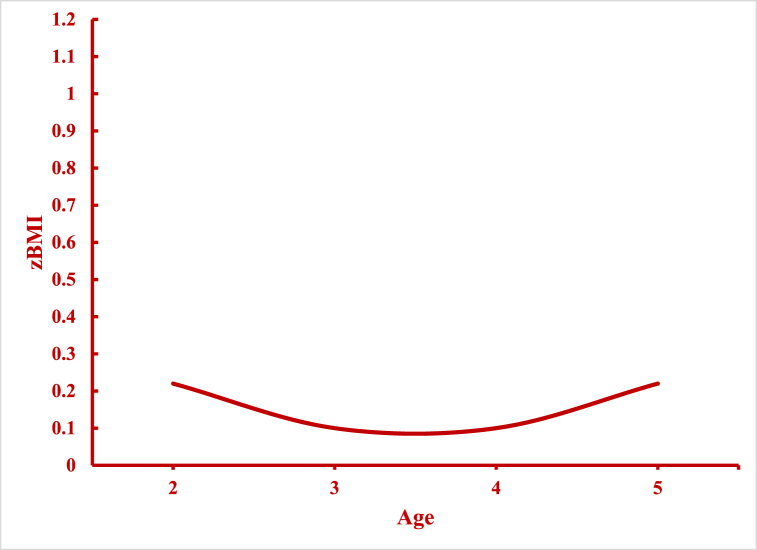

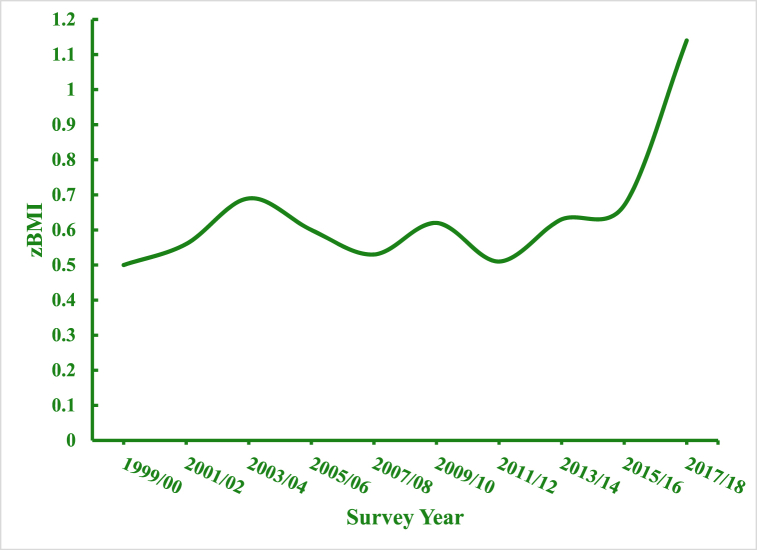

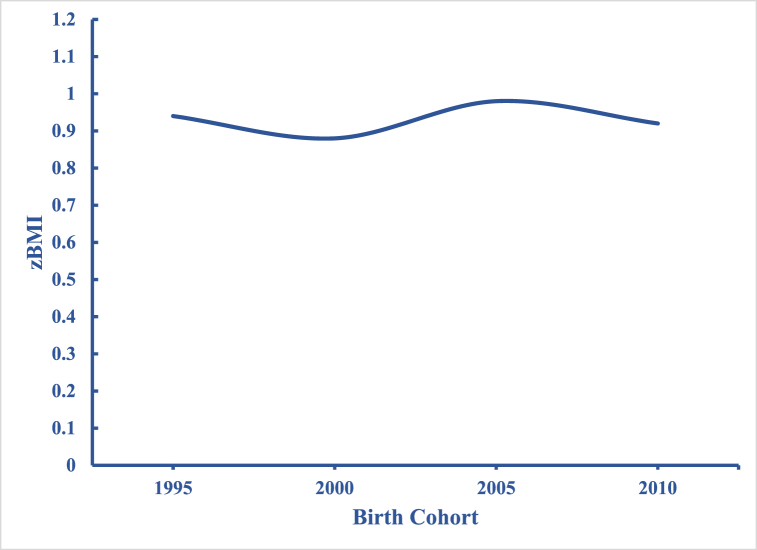
Overall Age, Period, and Cohort Effects on zBMI: NHANES 1999–2018 Fig. 1a. Age Effects, Fig. 1b. Period Effects, Fig. 1c. Cohort Effects.

In Fig. 1, we show the overall trends in zBMI estimated from Model 1. We see a clear non-linear relationship between zBMI and age (Fig. 1a). Initially, zBMI drops between 2 and 3 years of age, plateaus between 3 and 4 years of age, and rises between 4 and 5 years of age. In Fig. 1b, we present period effects estimated from Model 1. Specifically, children's zBMI are estimated for each year at the mean age and averaged over all birth cohorts (intercept + period-specific random-effect coefficients). zBMI trends are flat for nearly two decades, followed by a sharp increase in 2017/18. In Fig. 1c, we display estimated cohort effects from Model 1. zBMI is calculated at the mean age and averaged over all periods (intercept + cohort-specific random effect coefficients). The magnitude of cohort effects is rather small: zBMI fall between 0.88 and 0.98 SDs. Still, there are significant linear declines between the first and second cohort, followed by a sharp rise, and a rebounding decline among the last two cohorts. However, these results are strongly confounded by age and to a lesser extent period effects, making it difficult to draw any meaningful inferences from this pattern.

**Fig. 2:**
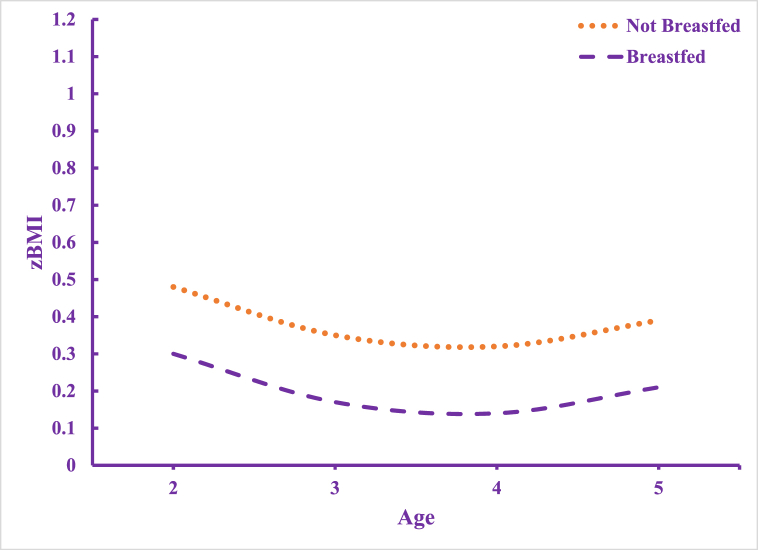
Predicted Age Variation in Sex on zBMI Note: Model 2 includes all independent variables and is graphed for the reference groups.

Model 2 results show that girls (−0.06 SDs; p < 0.05), those that identify as “Other” Race (−0.18 SDs; p < 0.001), mother's not smoking while pregnant (−0.15 SDs; p < 0.01), weighing less than five pounds at birth (−0.19 SDs; p < 0.01), and higher incomes (−0.01 SDs; p < 0.001) are associated with lower zBMI. Compared to those who identify as non-Hispanic white, Hispanics (0.16; p < 0.001), weighing more than or equal to nine pounds at birth (0.27 SDs; p < 0.001), not being breastfed (0.12 SDs; p < 0.001), and consuming higher than recommended amounts of protein (0.09 SDs; p < 0.05) are associated with higher zBMI. Crucially, these results show that individual-level effects highlighted in previous studies hold when level-2 heterogeneity in period and cohort effects are considered. Moreover, holding constant age and other social status indicators, children's zBMI significantly vary by cohort- and period-specific factors, as shown in the lower portion of Table 2. Specifically, the level-2 variance components show that most of the variance in zBMI is accounted for by individual-level characteristics. Still, significant variation exists by cohorts (0.00003; p < 0.05) and periods (0.00001; p < 0.05). The estimated average effect coefficients for periods reveal a particularly significant and negative effect for children surveyed in 2007/08 (−0.29; p < 0.10), 2011/12 (−0.31; p < 0.05), and 2015/16 (−0.15; p < 0.10). Model 2 further shows that the main age effect remains highly significant upon adjustment for all the above conditions. In Fig. 2, we display the predicted zBMI trajectories by sex estimated from Model 2. Girl and boy zBMI parallel one another but boy zBMI are consistently higher among all age groups (i.e., from 2 to 5 years of age).

**Fig. 3:**
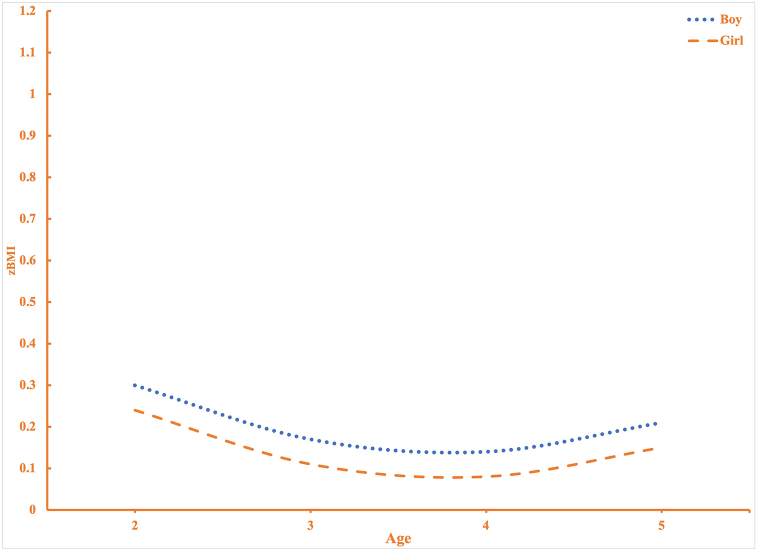
Predicted Age Variation in zBMI by Breastfeeding Status Note: Model 3 includes all independent variables and interaction effects and is graphed for the reference groups.

Model 3 is an additive model to Model 2 that includes a significant interaction effect between age and non-breastfed. Indeed, as non-breastfed children age, compared to those that are breastfed, there is an added zBMI increase of 0.06 SDs (p < 0.05) with every passing year. We graph this variation in Fig. 3 and show zBMI trajectories for breastfed relative to non-breastfed children. Non-breastfed children start out with higher zBMI and continue to have higher zBMI with every year increase in age.

## 4 Discussion

Consistent with previous studies of American children aged 2–5 years old, our current results indicate that zBMI for this age group reached a historic high [1,42]. Here we used a HAPC analysis to expand upon existing research and found that the changes in children's zBMI from 1999 to 2018 are driven primarily by the positive period effects rather than age and/or cohort effects. In other words, we are seeing the upward trend due to simultaneous changes in zBMI among the members of all cohorts, largely irrespective of age and generation. It should be mentioned that the highly pronounced positive period effect is offset by a nominal negative cohort effect (i.e., birth cohort membership), leading to a somewhat more gradual zBMI change. The slightly negative trend in birth cohort is harder to disentangle but changing prenatal [e.g., gestational weight gain (GWG)] and/or early postnatal conditions (e.g., breastfeeding duration) of successive cohorts may, in part, account for this finding [31,43]. For example, pre-pregnancy obesity has increased steadily over the years for all age groups and excessive GWG is a predictor for offspring obesity, but some antenatal diet-based and/or exercise-based lifestyle interventions that result in lower GWG also are associated with reduced risk of large-for-gestational-age neonate [[44], [45], [46]]. Also, published data indicate that maternal age at delivery has increased over time in the U.S [47]. This is important considering that breastfeeding rates at ≥24 months are higher among older (≥30 years) compared with those of younger (≤30 years) mothers, and breastfeeding is a protective factor for pediatric obesity [31,48]. Undoubtedly, however, children born to older mothers are at a higher risk for obesity throughout the life course [27]. It is clear that experts and leaders from academic, nonprofit, community, and government organizations must develop and implement nation-wide initiatives that focus on women of reproductive age because antenatal environmental factors such as poor nutrition contribute to epigenetic changes that lead to life-long “programming” of the fetus and influence offspring metabolism [49,50].

That being said, we observed a steep increase in zBMI from 1999 to 2018 due to the large positive period effects; however, *if* both period and cohort effects were positive they would amplify one another and create an even steeper zBMI change [51]. Because period effects, rather than differences in groups born at a specific time (i.e., cohort effects), account for almost all of the observed changes in zBMI, we need a broad socioeconomic, cultural, and environmental strategy to counteract the current obesogenic environment that influences preschoolers of all ages and generations [52,53]. Indeed, the longstanding rise in the prevalence of obesity among some of society's most vulnerable groups, pre-school aged children, is associated with upstream determinants (i.e., obesogens) such as the food system and culture in the U.S. that promotes the consumption of energy-dense diets with low nutritional value (e.g., ultra-processed foods, sugar-sweetened beverages, marketing of unhealthy foods), built environment (e.g., neighborhood walkability, access and proximity to food outlets and physical activity facilities), and technological advances and economic modernization (e.g., electronics) [49,[53], [54], [55]]. It is noteworthy that period effects also are driving the observed increase in the prevalence of obesity and mean BMI from 1999 to 2019 among American adolescents aged 14–18 years old [56]. Consequently, because time period-specific socioeconomic, cultural, and environmental changes create similar obesogenic contexts (e.g., public health policies or medical technology) for children of all ages, decision and policy makers must develop and employ ‘scaled up’ obesity prevention interventions (e.g., sugar-sweetened beverage excise tax, ban on child-directed unhealthy food advertising, urban farm tax credits) in order to reach large segments of children and achieve population-wide improvement [11,49,57,58]. The Special Supplemental Nutrition Program for Women, Infants, and Children (WIC), for example, is a federally funded health program that serves low-income, nutritionally-at-risk women, infants, toddlers, and children up to 5 years of age [59,60]. In April 2024, the U.S. Department of Agriculture published the *Final Rule: Revisions in the WIC Food Packages (2024)* in order to better align the WIC food packages with the *2020–2025 Dietary Guidelines for Americans* and to reflect recommendations from the Food and Nutrition Board within the National Academies of Science, Engineering, and Medicine [[61], [62], [63]]. Our presents findings about period and cohort effects support the idea that this updated public health policy is poised to have a tremendous impact on improving health and developmental outcomes for children, including the prevention of obesity, if WIC coverage rates improve, especially among children aged 1–4 years old, and evidence-based interventions such as the distribution of video content to be viewed at home are used to increase knowledge and affect behavior change among WIC participants [[64], [65], [66]].

Discussion around the origins and dynamics of the pediatric obesity epidemic demands scrutiny of empirical evidence that bridges levels of analysis, from individual to structural. In our present study, after accounting for period and cohort effects, we found U-shaped age effects. This means that the youngest (2-year-olds) and the oldest (5-year-olds) girls and boys are most affected by the temporal changes (i.e., period effects) that affect all age groups. It is clear that the obesogenic home environment uniquely influences the youngest children because caregivers dictate lifestyle-related practices such as healthy eating for the entire household [[67], [68], [69], [70]]. Indeed, there is a strong relationship between the BMI of family members [70]. Thus, the development of obesity among 2-year-olds may, in part, be explained by the caregiver approach to feeding during infancy, as well as maternal pre-pregnancy BMI and excessive GWG [[68], [69], [70], [71], [72], [73], [74], [75]]. Specifically, children in this age group who were never breastfed are more likely to have obesity compared to those children who were exclusively breastfed for six months, and weight gain is typically faster in formula-fed than in breastfed infants [[29], [30], [31],[76], [77], [78]]. Notably, the association between pre-pregnancy BMI and infant obesity is mediated by early-life feeding practices (e.g., inclusion of foods and beverages with added sugars) [79]. These effects, however, are complicated by many factors such as SES, as well as the association of obesity-related genes with early-life home environment [67,80]. It is important to note here that linear growth (i.e., length or height) is difficult to measure reliably in younger children, and measurement inaccuracies markedly affect the BMI value [81]. Similar to 2-year-old children, obesogenic food environment (e.g., availability of sugar sweetened beverages), maternal pre-pregnancy BMI, and excessive gestational weight gain also are associated with obesity in 5-year-olds [82,83]. Also, the magnitude of between-group (i.e., exclusively or predominantly breastfed vs. formula-fed infants) BMI differences is evident from age 7 months and increases with age [84]. Additionally, caregiver rules around electronic devices (i.e., screen time) shape older children's (e.g., 5 years of age) body weight trajectories [85].

Here we show that individual-level effects on child obesity described in previous studies hold when level-2 heterogeneity in period and cohort effects are considered. For example, within populations, the prevalence of obesity is higher among boys compared to girls [1,86,87]. Similarly, the variation in body weight associates with race/ethnicity and SES, wherein Hispanic children and those living in low-income households are disproportionally burdened by obesity [88,89]. Published data indicate that mothers with fewer social, educational, and economic resources are more likely to follow non-recommended infant feeding practices such as predominant formula feeding, early introduction of solid foods, and using food as a reward [[90], [91], [92], [93], [94], [95]]. Related, those with lower SES have higher pre-conception BMI and are more likely to smoke cigarettes during pregnancy [96]. All of the above mentioned factors shape children's body weight trajectories [[93], [94], [95], [96]]. For example, maternal cigarette smoking during pregnancy increases the odds of rapid infant weight gain and childhood overweight/obesity, independent of maternal pre-pregnancy BMI and genetic predisposition to adiposity [28,[97], [98], [99]]. Importantly, smoking cessation may reduce the risk of rapid infant zBMI gain and childhood overweight and obesity [[100], [101], [102]].

We are the first to show that pediatric zBMI rose from 1999 to 2018 because the typical child between 2 and 5 years of age in all cohorts is gaining weight simultaneously. Still, our study is not without limitations. The use of repeated cross-sectional data restricts our ability to offer any causal explanations [36]. Crucially, however, the only way to separate the putative population-level mechanisms that are generating change is to track multiple cohorts’ experiences over time, which can be done only with repeated cross-sectional data [40]. Moreover, despite the rich individual-level descriptive data available in NHANES, earlier survey waves lack important confounders (e.g., physical activity for this age group was introduced in 1999/2000 and sugar intake in 2003/2004), thereby limiting our ability to use earlier waves of data. Related, household smoking status is associated with pediatric obesity, but we are unable to include this measure due to significant sample size reductions that may induce estimate bias [40,103]. All APC models have certain strengths and weaknesses [104]. Some argue, for example, that the HAPC model favors period explanations as a direct function of the data structure (i.e., data is collected by survey waves and not birth cohorts), thus resulting in a wider range of periods than cohorts [[105], [106], [107]]. In our current study, the likelihood that the random effects portion is artificially inflated is reduced since we use 10 waves of data collected between 1999 and 2018, along with four 5-year groupings of cohorts for children born between 1995 and 2016 [108,109]. Still, because the estimated results may depend on the specific constraints chosen, we also performed robustness checks using 2-year cohort intervals and alternative functional forms of age. Results indicate that these modifications do not substantively change the findings. Finally, BMI is a simple measure but reasonably good for diagnosing pediatric obesity, especially when height and weight measures are collected objectively [36,110,111]. Even with these limitations, our study provides a substantial first step necessary to isolate and eliminate population-level increases in already-too-high pediatric zBMI.

It is alarming that the U.S. has never met the *Healthy People 2020* goal of 9.6 % obesity prevalence among children 2–5 years of age [16,35]. Unfortunately, *individual*-level approaches (e.g., lifestyle modifications) that only focus on diet *or* physical activity have not produced meaningful reductions in BMI or zBMI among children aged 0–5 years [32]. Yet, there is ground for optimism because interventions that involve a combination of lifestyle changes (e.g., energy intake reduction *and* physical activity increase *and* sedentary activity reduction) have the potential to reduce the risk of obesity (BMI and zBMI) in this age group [32]. Important for our current study, multi-pronged *population*-level approaches such as federal assistance programs, SSB taxes, and community-wide interventions (e.g., early care centers, school, after-school clubs) have the greatest impact on improving preschoolers' weight status [109]. Related, we need national guidelines to limit preschool children's daily media use because watching television/videos and computer use are associated with obesity and adiposity [82,110]. Also, because obesity follows a social gradient (e.g., children who experience poverty are 1.6 times more likely to be diagnosed with obesity), policy makers must consider socioeconomic deprivation in order to eliminate the immense disparity in the rates of childhood obesity among racial/ethnic groups [49,[111], [112], [113], [114], [115]]. Taken together, per the World Health Organization framework, “universal prevention” (i.e., entire community) strategies for healthy weight promotion must be combined with “selective prevention” (i.e., at-risk groups) and/or “targeted prevention” (i.e., at-risk individuals) approaches in order to reverse the pediatric obesity epidemic [116].

## Data availability statement

NHANES is publicly available at cdc.gov.

## CRediT authorship contribution statement

**Ashley W. Kranjac:** Writing – review & editing, Writing – original draft, Validation, Supervision, Software, Methodology, Investigation, Formal analysis, Data curation, Conceptualization. **Dinko Kranjac:** Writing – review & editing, Writing – original draft, Supervision, Resources, Project administration, Investigation, Conceptualization. **Roxanne I. Aguilera:** Writing – review & editing, Resources, Investigation, Data curation.

## Declaration of competing interest

The authors declare that they have no known competing financial interests or personal relationships that could have appeared to influence the work reported in this paper.

## Footnotes

1 Missing cases exist for various variables in every survey. Caloric intake (n = 3693) and physical activity (n = 4021) measures have the largest number of missing cases. Listwise deletion reduced our sample size but resulted in a final sample of participants who have data on all the variables in the analysis. We retained a sufficient number of cases for the analyses, and using the same sample across models aides in the comparison of model fit.
